# FAM201A encodes small protein NBASP to inhibit neuroblastoma progression via inactivating MAPK pathway mediated by FABP5

**DOI:** 10.1038/s42003-023-05092-7

**Published:** 2023-07-12

**Authors:** Mujie Ye, Runnan Gao, Shiyu Chen, Jianan Bai, Jinhao Chen, Feiyu Lu, Danyang Gu, Xiaoting Shi, Ping Yu, Ye Tian, Qiyun Tang, Kuiran Dong

**Affiliations:** 1 Department of Geriatric Gastroenterology, Neuroendocrine Tumor Center, Jiangsu Province Hospital, The First Affiliated Hospital of Nanjing Medical University, Institute of Neuroendocrine Tumor, Nanjing Medical University, Nanjing, China; 2 Department of Pediatric Surgery, Children’s Hospital of Fudan University, National Children’s Medical Center, Shanghai, China; 3 Department of Biochemistry and Molecular Biology, Research Center for Birth Defects, Institutes of Biomedical Sciences, Key Laboratory of Metabolism and Molecular Medicine, Ministry of Education, School of Basic Medical Sciences, Fudan University, Shanghai, China

**Keywords:** Cancer epigenetics, Paediatric cancer

## Abstract

Increasing evidence indicates that long non-coding RNA (lncRNA) is one of the most important RNA regulators in the pathogenesis of neuroblastoma (NB). Here, we found that FAM201A was low expressed in NB and a variety of gain and loss of function studies elucidated the anti-tumor effects of FAM201A on the regulation of proliferation, migration and invasion of NB cells. Intriguingly, we identified the ability of FAM201A to encode the tumor-suppressing protein, NBASP, which interacted with FABP5 and negatively regulated its expression. In vivo assays also revealed NBASP repressed NB growth via inactivating MAPK pathway mediated by FABP5. In conclusion, our findings demonstrated that NBASP encoded by FAM201A played a tumor-suppressor role in NB carcinogenesis via down-regulating FABP5 to inactivate the MAPK pathway. These results extended our understanding of the relationship of lncRNA-encoded functional peptides and plasticity of tumor progression.

## Abstract (web summary)

The long non-coding RNA FAM201A encodes a short peptide NBASP that suppresses neuroblastoma carcinogenesis by down-regulating FABP5 to inactivate the MAPK pathway.

## Introduction

Neuroblastoma (NB) is the most common extracranial pediatric solid tumor that originates from the developing peripheral sympathetic nervous system, accounting for about 8% of all pediatric cancers, and usually affecting children within the first 5 years of life^1–3^. With improvements of effective interventions and targeted therapies, long term survival in children, especially high risk patients suffering chemo-resistant relapse, is still <40%^3^. NB presents with remarkable heterogeneities in clinical phenotype, localization, and genetics involving abnormalities at the genome, epigenome, and transcriptome levels^4–6^. Due to the complexity and heterogeneity of NB, a comprehensive understanding of the etiology controlling the tumorigenesis of NB may contribute to a better understanding of the molecular pathogenesis of NB, thus, providing the basis for effective diagnoses and biological therapies.

In addition to the identification of several genomic alterations in NB over the past few decades, showing a wide range of chromosomal abnormalities and genetic abnormalities, dysregulation of the non-coding portion of the genome provide additionally promising mechanisms regarding NB initiation and progression, especially with those involving long non-coding RNAs (lncRNAs)^7,8^. LncRNAs are a group of RNAs longer than 200 nucleotides that regulate gene expression at transcriptional and post-transcriptional levels^9^. By using bioinformatics and high throughput methods, recent studies have revealed that a dysregulated lncRNA profile was widely involved in the pathogenesis of tumors. Surprisingly, a growing number of studies have found that some lncRNAs contained functional small open reading frames (smORFs < 300 nt)^10–12^, encoding functional micropeptides that played a key role in the development and progression of cancer^13,14^. Liu et al. found that SMIM30, a conserved 59 amino acid (aa) peptide encoded by LINC00998, promoted hepatocellular carcinoma (HCC) tummorigenesis^13^. Yan et al. discovered that oncopeptide RBRP (an RNA-binding regulatory peptide) encoded by LINC00266-1 strengthened m6A recognition of targets to exert oncogenetic functions^14^. Applying these peptides encoded by lncRNAs as therapeutic targets in cancer is increasingly promising, but the precise role of these peptides in the initiation and development of NB remains unclear.

The lncRNAs are a family with sequence similarity, with the 201 member A (FAM201A) reported to act as an oncogene in lung cancer by interacting with miR-7515, miR-101, and miR-370^15–17^, breast cancer via miR-186-5p/*TNKSBP1* axis^18^, and hepatic cancer^19^, and it might be a potential therapeutic target. In contrast, in our study, we discovered that FAM201A was down-regulated in NB tissues, and we therefore showed the tumor suppressor role in NB. Furthermore, we found that FAM201A encoded a conserved 155 amino acid peptide named NBASP (neuroblastoma-associated small protein). Moreover, NBASP negatively regulated fatty acid-binding protein 5 (FABP5) thus affected fatty acid metabolism to act as tumor-suppressing effect. Overall, our findings provided valuable insights into the molecular mechanism of NB tumor epigenetic alterations, and suggested that NBASP could be a critical target for the treatment of NB.

## Results

### FAM201A over-expression inhibits NB cell proliferation, migration and invasion

**Fig. 1:**
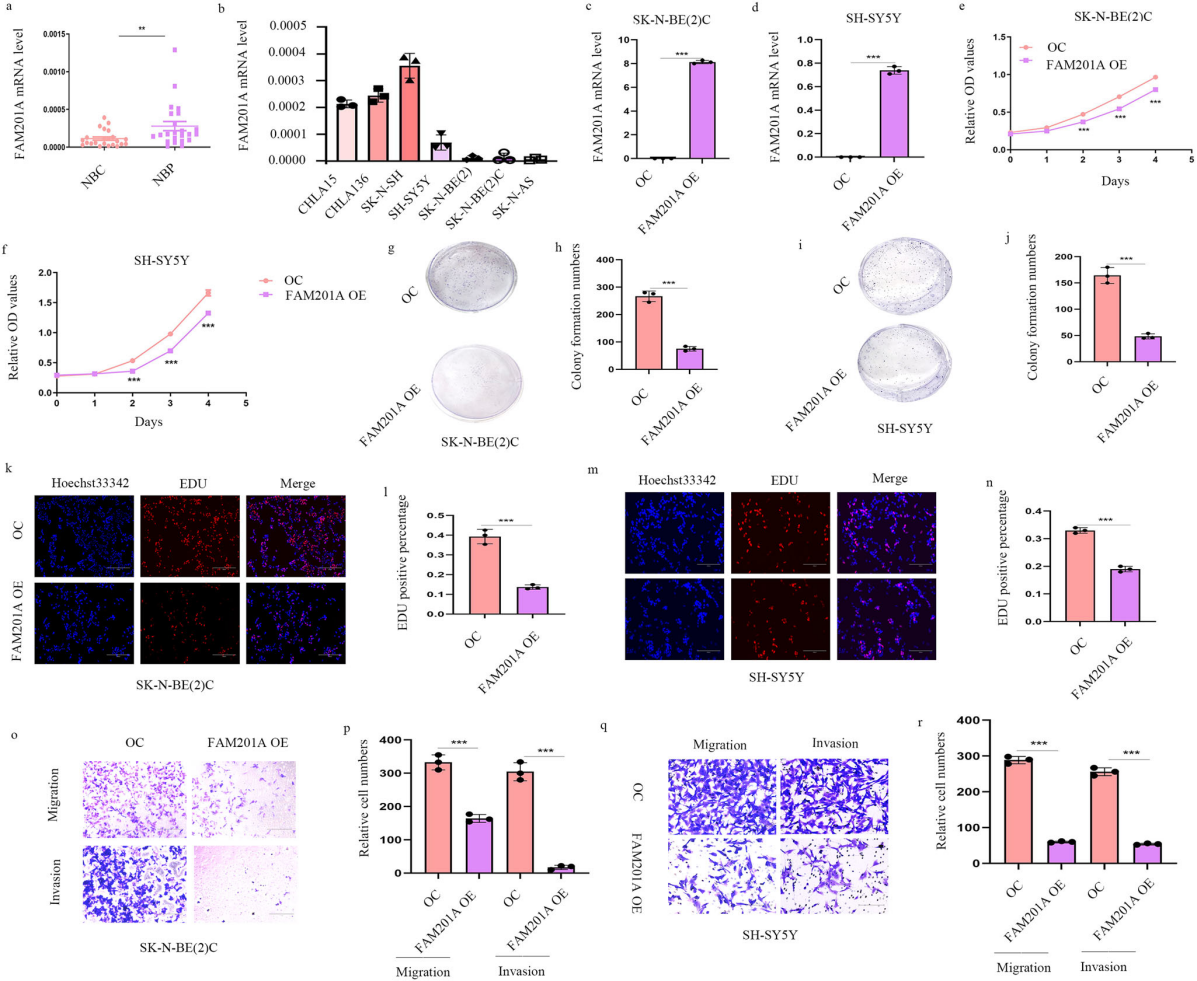
FAM201A over-expression represses NB cell proliferation, migration and invasion. **a** Decreased expression of FAM201A expression was detected in NB tissues (NBC, *n* = 23) and adjacent normal tissues (NBP, *n* = 23) by Q-PCR analyses; **b** Q-PCR analysis showed the expressions of FAM201A in CHLA15, CHLA136, SH-N-SH, SH-SY5Y, SK-N-BE(2), SK-N-BE(2)C, and SK-N-AS cell lines; **c** and **d** SK-N-BE (2)C and SH-SY5Y cells were transfected with FAM201A over-expression vectors and a empty vector (OC represents over-expression control vectors), and the over-expression effect was verified by Q-PCR analysis; **e** and **f** Over-expression of FAM201A inhibited both SK-N-BE (2)C and SH-SY5Y cell proliferation, as shown by CCK8 assays; **g**–**j** The number of colonies decreased dramatically after over-expression of FAM201A in the SK-N-BE (2)C and SH-SY5Y cells; **k**–**n** Over-expression of FAM021A decreased SK-N-BE (2)C and SH-SY5Y cell proliferation, as shown by EdU assays, magnification, ×200, scale bar, 300 μm; **o**–**r** Transwell migration and matrigel invasion assays were used to determine the cell migration and invasion capabilities of SK-N-BE (2)C and SH-SY5Y cells transfected with the over-expression of FAM201A and empty vectors. Shown are quantitation of cell numbers from three independent experiments, magnification, ×100, scale bar, 300 μm. Results in (**c**–**r**) are mean ± SD (*n* = 3). Statistical analysis was done by Student’s *t* test. (**p < 0.01, ***p < 0.001).

To identify the roles of the FAM201A in the progression of NB, we examined its RNA levels in 23 pairs of NB and adjacent normal tissue in our cohort. The Q-PCR results showed that FAM201A was reduced in NB tumor tissues, when compared with non-tumor samples (Fig. 1a). Next, we measured FAM201A levels in seven NB cell lines (Fig. 1b). To clarify the potential role of FAM201A in NB tumorigenesis, we constructed vectors stably over-expressing FAM201A using the pCDH plasmid in SH-SY5Y and SK-N-BE (2)C cells with a low endogenous level of FAM201A. Q-PCR determined the over-expression efficiency in two NB cell lines (Fig. 1c,d). And over-expression in FAM201A resulted in decreased cell viability, as shown by the CCK-8 and colony formation assays (Fig. 1e–j). In accordance with the results from the CCK-8 assay, EdU incorporation assays were conducted to determine similar adverse effects of FAM201A on cell proliferation (Fig. 1k–n). These results suggested that over-expressing FAM201A decelerated the growth of SH-SY5Y and SK-N-BE (2)C cells. In addition, transwell assays showed that over-expression of FAM201A decreased NB cell migration and invasion in vitro (Fig. 1o–r). Together, these results showed that FAM201A had inhibitive effects on NB cell proliferation, migration and invasion.

### Loss of FAM201A triggers NB cell proliferation, migration and invasion

**Fig. 2:**
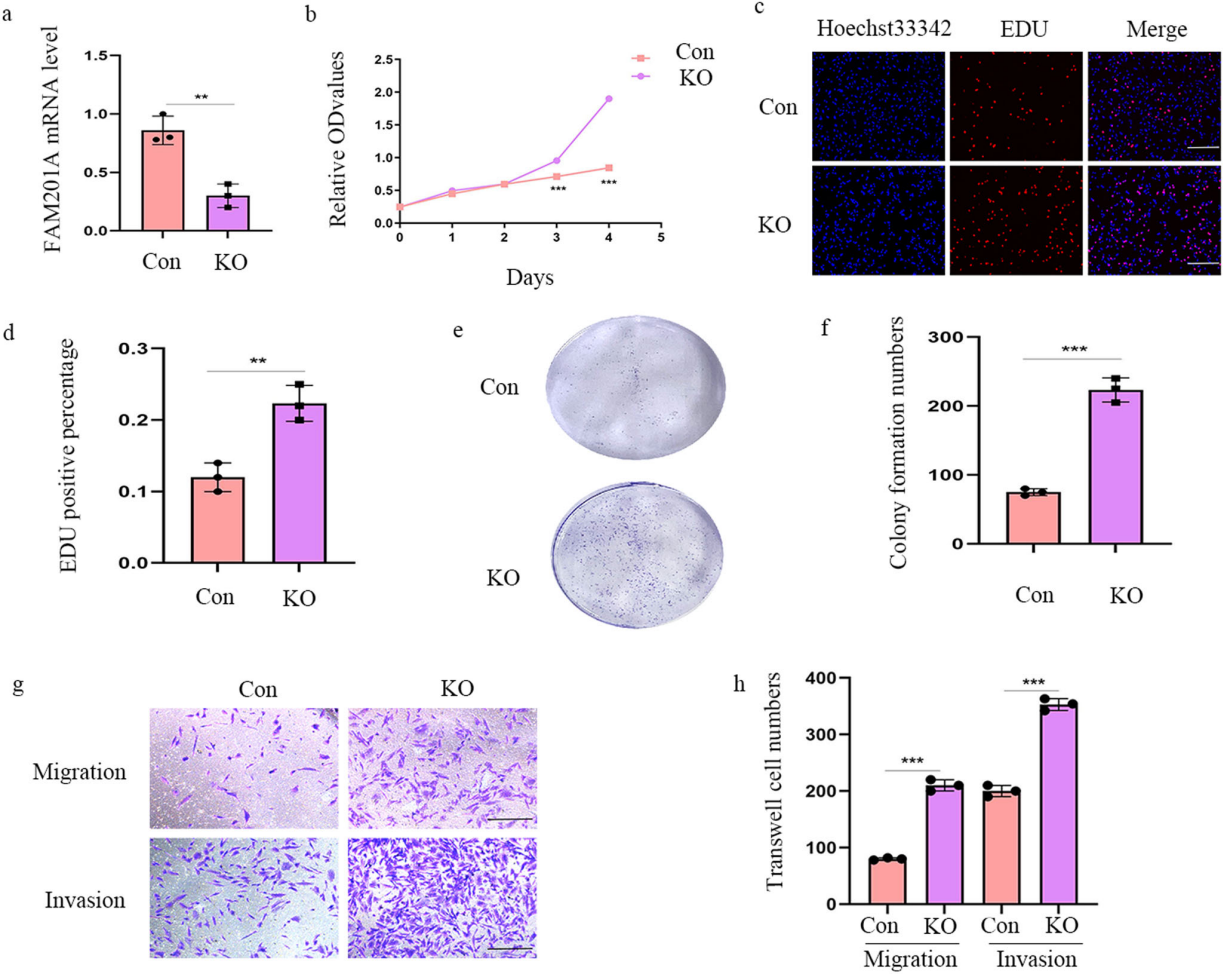
Loss of FAM201A triggers NB cell proliferation and metastasis. **a** The effects of CRISPR Cas9 knockout of FAM201A expression; **b** Knockout of FAM201A on proliferation in SK-N-SH cells was determined by CCK8 assays; **c** and **d** Knockout of FAM021A accelerated SK-N-SH cell proliferation, as shown by EdU assays, magnification, ×200, scale bar, 300 μm; **e** and **f** The colonies increased after knockout of FAM201A in SK-N-SH cell lines; **g** and **h** Transwell migration and matrigel invasion assays were used to determine the cell migration and invasion capabilities of knockout of FAM201A in SK-N-SH cells. Shown are quantification of cell numbers from three independent experiments, magnification, ×100, scale bar, 300 μm. Results in (**a**–**h**) are mean ± SD (*n* = 3). Statistical analysis was done by Student’s *t* test. (^**^p < 0.01, ^***^p < 0.001).

To further characterize the anti-tumor potential effects of FAM201A, we used CRISPR Cas9 to knockout the endogenous levels of FAM201A in SK-N-SH cells, which had highest FAM201A expression (Fig. 2a). The deficiency of FAM201A accelerated the proliferation measured by CCK-8 and EdU incorporation assays in NB cells (Fig. 2b–d). Consistently, colony formation assays confirmed the proliferative role of decreased expression of FAM201A in NB cells (Fig. 2e,f). Moreover, transwell experiments showed that knockout of FAM201A induced NB cell mobility (Fig. 2g–h). Overall, these results verified the anti-tumor role of FAM201A in NB cells.

### FAM201A encodes a small endogenous peptide expressed in NB cells

**Fig. 3:**
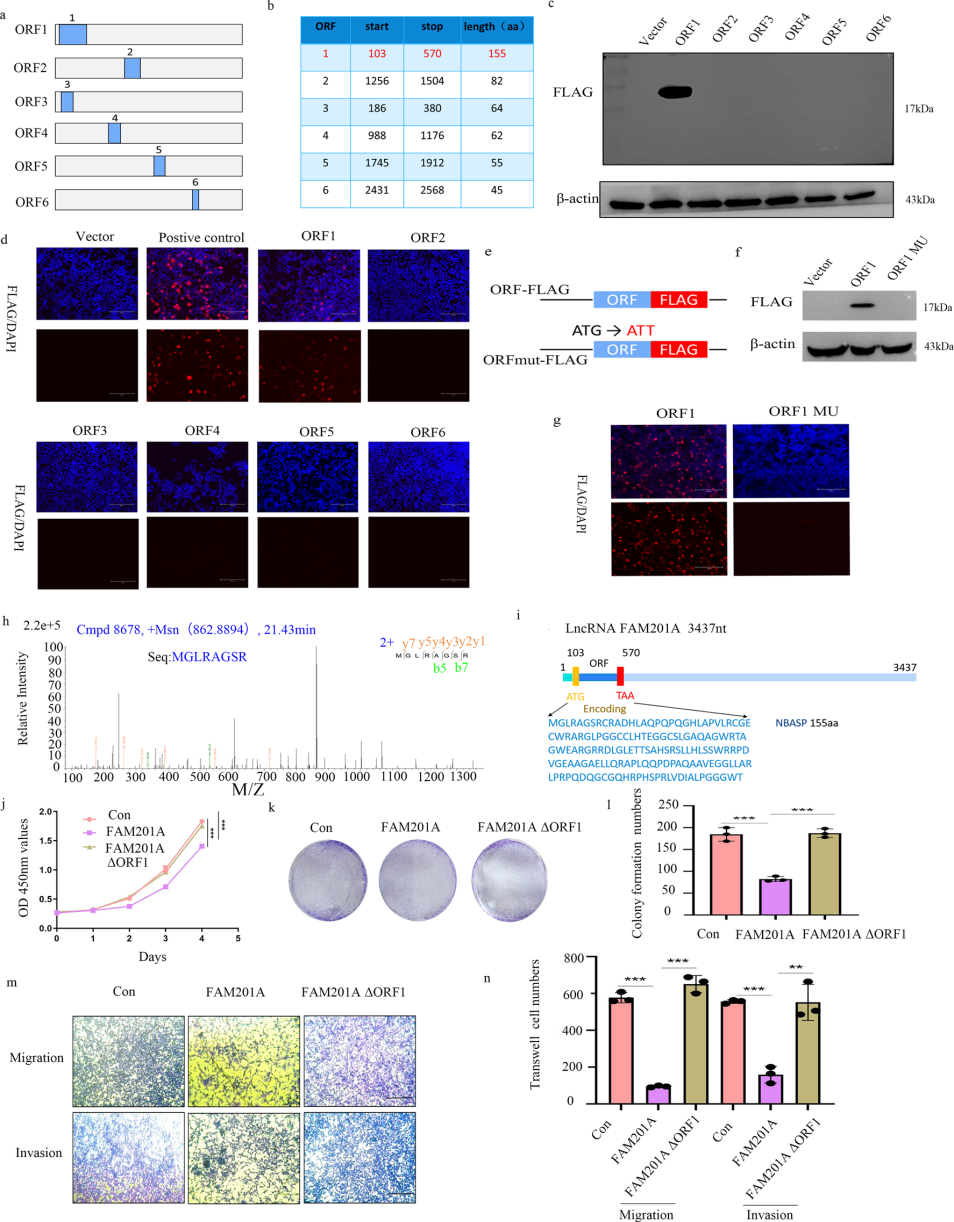
FAM201A encodes a small endogenous peptide expressed in NB cells. **a** Data from the Coding Potential Assessment Tool database predicted six open reading frames (ORFs) using the ORF finder; **b** The start, stop location, and the length of these predicted ORFs are shown; **c** The 293 T cells were cloned with a FLAG epitope tag within the C-terminus (Flag knock-in) before the stop codons of the six candidate ORFs of FAM201A, and a empty vector (negative control). Only the ORF1 could encode peptides detected by western blotting; **d** Only ORF1-encoded peptides further confirmed by immunofluorescence staining, positive control represents a coding gene with FLAG; **e** A mutation of ATG (ΔATG, G-T) containing the FLAG fusion sequence was constructed as shown; **f** and **g** The expression of the ORF1-encoded peptide was abolished by mutating the ATG codons of ORF1, as evidenced by western blotting and immunofluorescence analysis, magnification, ×200, scale bar, 200 μm; **h** and **i** Results from mass spectrometry analysis confirmed the existence of the endogenous peptide encoded by ORF1 in NB cells; **j** CCK8 assay of FAM201A over-expression, FAM201A over-expression with ORF1 depletion, and control groups; **k** and **l** Colony formation experiments and statistics of FAM201A over-expression, FAM201A over-expression with ORF1 depletion and control groups; **m** and **n** Transwell assay of FAM201A over-expression, FAM201A over-expression with ORF1 depletion, and control groups, magnification, 100, scale bar, 300 μm. Results in (**j**–**n**) are mean ± SD (*n* = 3). Statistical analysis was done by Student’s *t* test. (^**^p < 0.01, ^***^p < 0.001).

To identify the mechanism of FAM201A participation in NB progression, we identified the regulation pattern of this lncRNA. Data from the Coding Potential Assessment Tool (CPAT) database indicated that FAM201A might encode a functional peptide. Although FAM021A was initially described as non-coding, six open reading frames (ORFs) were predicted by the ORF finder (Fig. 3a,b). Next, to determine whether FAM201A was translated into a peptide, we cloned a FLAG epitope tag within the C-terminus before the stop codons of the selected ORF of FAM201A. Only a peptide encoded by ORF1 yielded a peptide of 17 kDa in 293 T cells, detected by western blotting (Fig. 3c), which was further confirmed by immunofluorescence (Fig. 3d). To further evaluate the encoding capacity of ORF1 of FAM201A in peptide translation, a mutation of ATG (ATG to ATT) containing a FLAG fusion sequence was constructed (Fig. 3e). Western blotting and immunofluorescence analyses showed that expression of the ORF1-encoded peptide was abolished by mutating the ATG codons of ORF1 (Fig. 3f,g).

To verify whether this peptide could be endogenously expressed, we used mass spectrometry analysis to confirm the existence of the endogenous peptide. As expected, results from mass spectrometry confirmed the existence of peptide encoded by ORF1 in NB cells (Fig. 3h,i). In addition, we constructed a mutant of FAM201A by depletion of ORF1. Cell function assay demonstrated that depletion of ORF1 in FAM201A resulted in loss of anti-cancer effect of FAM201A by CCK8, colony formation and transwell assays(Fig. 3j–n). To the best of our knowledge, it was a newly discovered putative peptide, so we named it neuroblastoma-associated small protein (NBASP).

### NBASP encoded by ORF1 of FAM201A suppresses proliferation, migration and invasion in NB cells

**Fig. 4:**
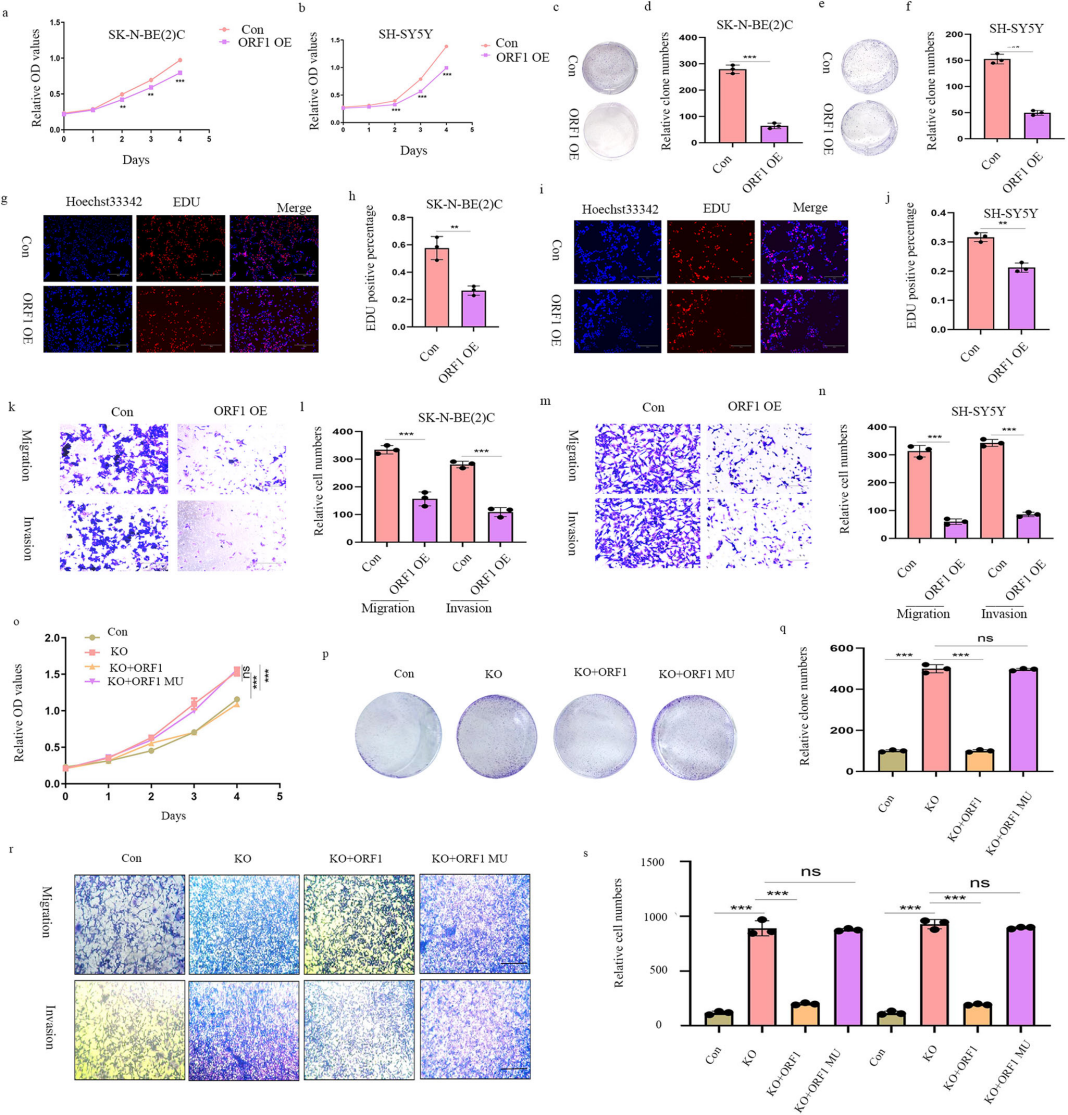
NBASP inhibits NB proliferation, migration and invasion in vitro. **a** and **b** Over-expression of ORF1 in both SH-SY5Y and SK-N-BE (2)C cells inhibited proliferation, as shown by the CCK8 assay; **c**–**f** Colony formation showed that over-expression of ORF1 decreased the proliferation in SH-SY5Y and SK-N-BE (2)C cells; **g**–**j** The EdU staining further confirmed that a high level of NBASP suppressed the viability of NB cells, magnification, ×200, scale bar, 300 μm; **k**–**n** Cell counts of the lower chamber in ORF1 over-expression groups were found to be decreased, when compared with the control group, magnification, ×100, scale bar, 300 μm. **o** CCK8 assay of FAM201A knockout, ORF1 restore, ORF1 mutation at start codon(ATG to ATT) restore, and control groups; **p** and **q** Colony formation experiments and statistics of FAM201A knockout, ORF1 restore, ORF1 mutant restore, and control groups; **r** and **s** Transwell assay of FAM201A knockout, ORF1 restore, ORF1 mutant restore, and control group, magnification, ×100, scale bar, 300 μm. Results in (**a**–**s**) are mean ± SD (*n* = 3). Statistical analysis was done by Student’s *t* test. (^**^p < 0.01, ^***^p < 0.001).

To gain insight into the functional role of NBASP in NB cells, we established vectors stably over-expressing ORF1 using the pCDH plasmid in SH-SY5Y and SK-N-BE (2)C cells. Similar to FAM201A, cell proliferation, as shown by the CCK-8 and colony formation assays, was inhibited in SH-SY5Y and SK-N-BE (2)C cells with over-expression of ORF1 (Fig. 4a–f). EdU staining further confirmed that a high level of NBASP suppressed the viability of NB cells (Fig. 4g–j). Transwell assays showed that NBASP was associated with the metastatic behaviors of NB cell lines. Cell counts of the lower chambers in ORF1 over-expression groups were found to be decreased, when compared with the control group (Fig. 4k–n). To further investigate ORF1 roles in NB, we performed several restored experiments. CCK8 and colony formation assay showed ORF1 inhibited cell proliferation induced by FAM201A knockout while ORF1 mutant failed to restored the effect by FAM201A knockout (Fig. 4o–q). Moreover, transwell results also indicated ORF1, not its mutant, rescued inhibition cell migration and invasion of FAM201A (Fig. 4r,s). Together, these results indicated that over-expressed ORF1 resulted in proliferation and metastasis suppression, similar to FAM201A itself on NB cell lines and ORF1 could restore the role of FAM201A knockout in promoting tumor.

### Small protein NBASP interacts with FABP5 and reduce its expression via the ubiquitin proteasome pathway

**Fig. 5:**
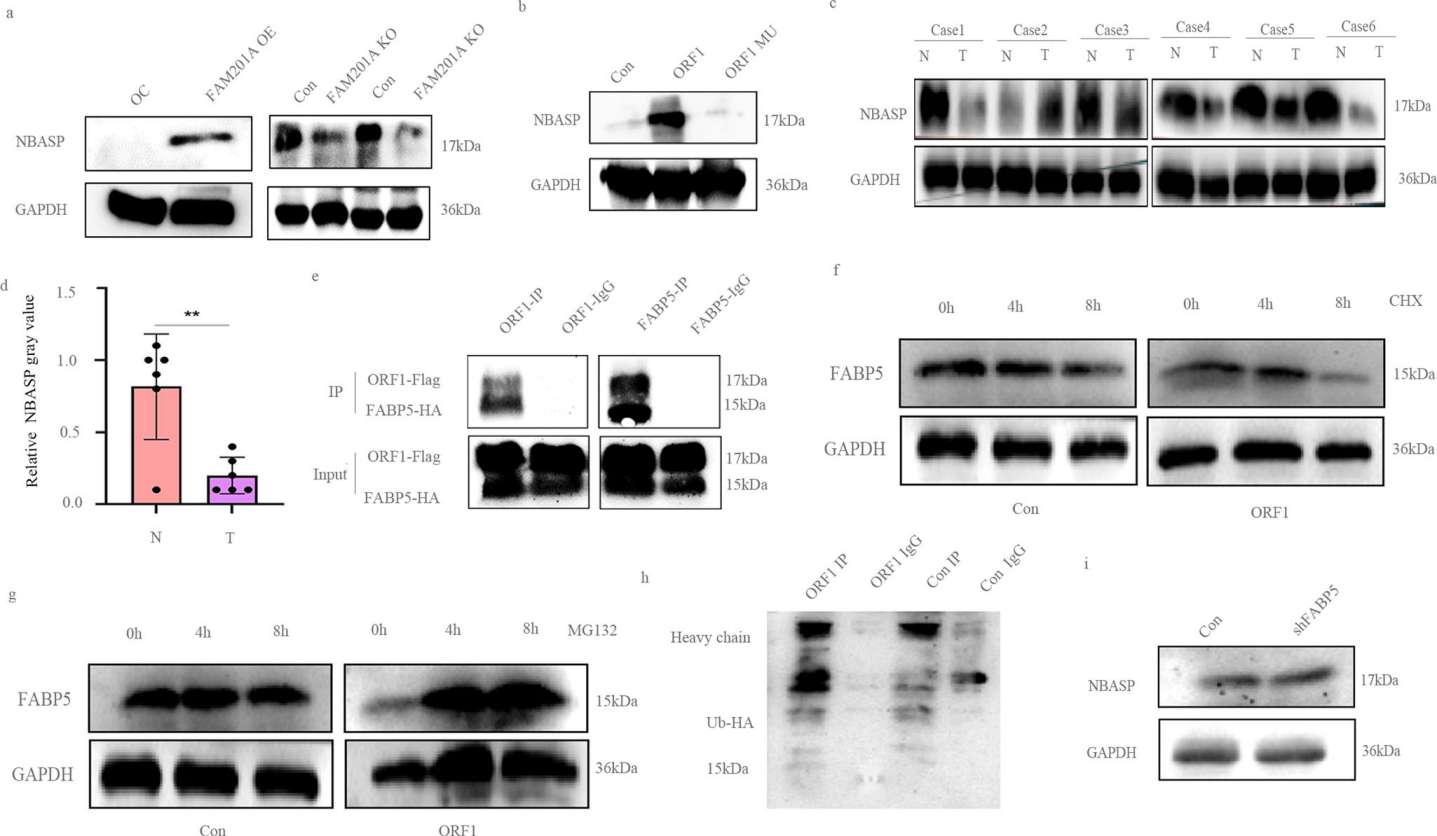
NBASP regulates FABP5 via ubiquitin proteasome pathway. **a** and **b** Western blotting of NBASP protein expression upon over-expression and knockout of the FAM201A, ORF1, and ORF1-mutant in NB cells; **c** and **d** Down-regulation of NBASP was confirmed in tumor tissues when compared with adjacent normal tissues (*n* = 6) at the protein level by western blotting; **e** The interaction between NBASP and FABP5 was confirmed by a co-immunoprecipitation assay; **f** Western blotting was performed to detect FABP5 level in ORF1 over-expression and control group after 10 μm CHX treatment for 0 h, 4 h, 8 h; **g** Western blotting was performed to detect FABP5 level in ORF1 over-expression and control group after 10 μm MG132 treatment for 0 h, 4 h, 8 h; **h** Co-IP showed ORF1 over-expression bound to more ubiquitin than control group; **i** Down-regulation of FABP5 has no effect on NBASP. Statistical analysis was done by Student’s *t* test. (**p < 0.01).

To better study NBASP, we customized an antibody and executed several western blotting assay. These results demonstrated over-expression of FAM201A induced NBASP level while knockout FAM201A decrease NBASP level (Fig. 5a). Similarly, transfection of ORF1 increased NBASP expression but ORF1 mutation had not the function (Fig. 5b). Furthermore, western blotting also showed down-regulation of NBASP in NB tissues compared to adjacent normal tissues (Fig. 5c, d). To further investigate the mechanism by which the NBASP peptide interfered with NB development, we performed immunoprecipitation combined with mass spectrometry analysis. It showed that the candidate protein fatty acid-binding protein 5 (FABP5) interacted with NBASP. A previous study reported that FABP5, as a tumorigenic protein, was up-regulated in MYCN amplified NB cells, to promote proliferation and migration^20^. Based on these findings, we postulated that NBASP interacted with FABP5, involving the development of NB. Consistent with this possibility, the Co-IP assay showed interactions between NBASP and FABP5 (Fig. 5e). Immediately after, we used cycloheximide(CHX) and MG132 treated NB cells. After 8 h treatment, ORF1 over-expression has a FABP5 decrease compared to control group (Fig. 5f). Moreover, FABP5 was increase in ORF1 over-expression group after 4 h MG132 treatment (Fig. 5g). After 24 h of ubiquitin transfection, Co-IP assay of FABP5 and ubiquitin was done and it indicated more ubiquitin molecules bound to FABP5 in ORF1 over-expression group than control group (Fig. 5h). Conversely, FABP5 knockdown did not affect the expression of NBASP (Fig. 5i).

### FABP5 is highly expressed in NB and acts as oncogene in NB

**Fig. 6:**
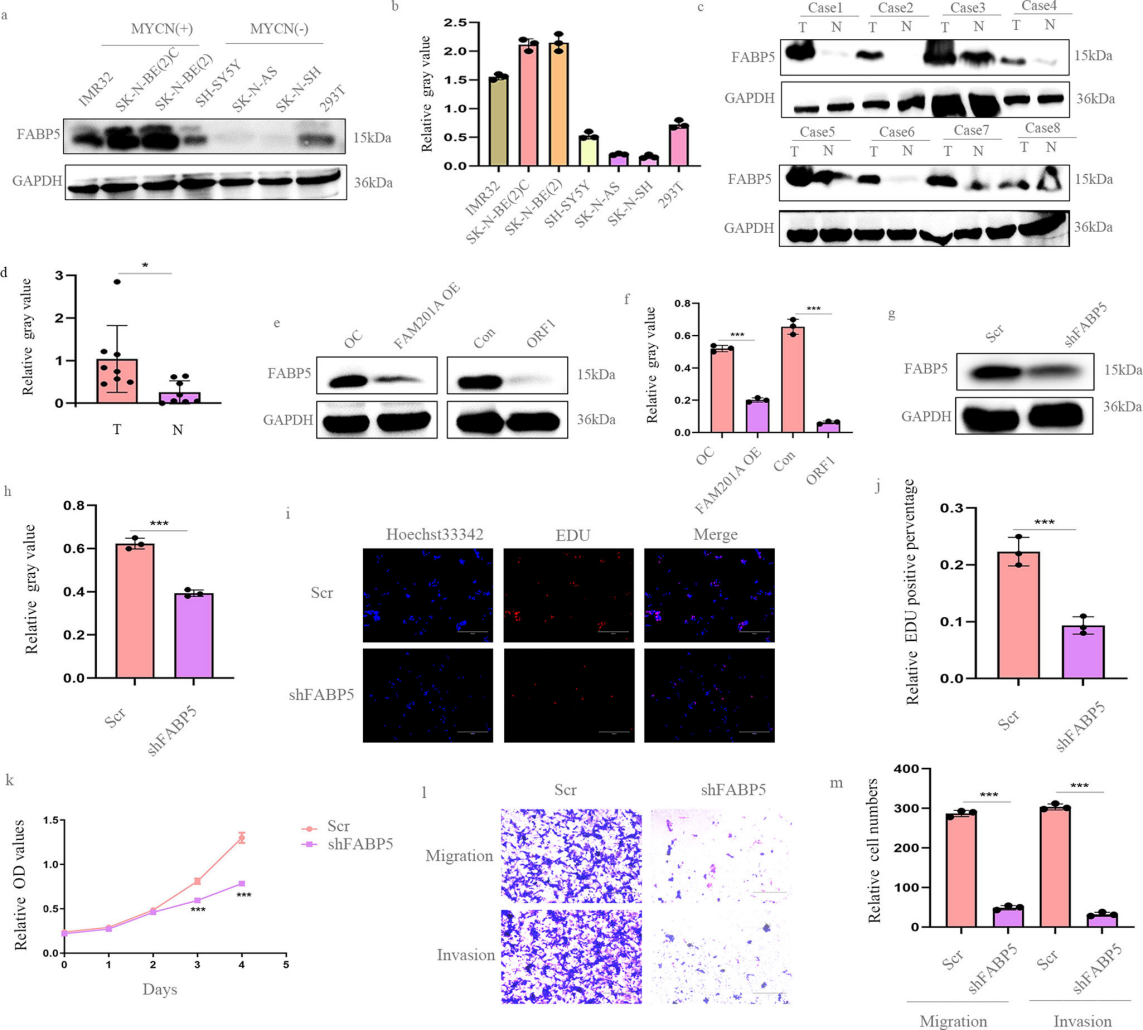
FABP5 is highly expressed in NB and acts as oncogene in NB. **a** and **b** FABP5 was up-regulated in NB cell lines with MYCN amplification, when compared with non-MYCN amplified NB cell lines and 293 T cell lines (negative control), as showed by western blotting. Shown is the quantitation of relative gray values; **c** and **d** Up-regulation of FABP5 was confirmed in tumor tissues when compared with normal tissues (*n* = 8) at the protein level by western blotting; **e** and **f** Western blotting of FABP5 protein expression upon over-expression of FAM201A and ORF1 in NB cells, which was quantified by relative gray values; **g** and **h** The knockdown efficiency of FABP5 was evaluated and quantified by western blotting analysis in NB cells; **i**–**k** The effects of shFABP5 in NB cells were determined by EdU and CCK8 assays, magnification, ×200, scale bar, 300 μm; **l** and **m** A decrease in cell number in the transwell assay was observed in the FABP5 knockdown groups, magnification, ×100, scale bar, 300 μm. Results in (**a**–**m** except **c**,**d**)are mean ± SD (*n* = 3). Statistical analysis was done by Student’s *t* test. (^*^p < 0.05, ^***^p < 0.001).

As shown in our study, we found that total protein levels of FABP5 were up-regulated in NB cell lines with MYCN amplification, when compared with the non-MYCN amplified NB cell lines (Fig. 6a,b). These results were further confirmed in NB tissues by western blotting. At the protein level, FABP5 was up-regulated in NB tissues compared to regular counterparts (Fig. 6c,d). Furthermore, western blotting demonstrated up-regulation of FAM201A or ORF1 decreased FABP5 in protein level (Fig. 6e,f). To further characterize the role of FABP5 in NB, we silenced its expression in NB cells. The knockdown efficiency of FABP5 was evaluated and quantified by western blotting in NB cells (Fig. 6g,h). EdU incorporation and CCK-8 assays were conducted to determine the effects of FABP5 on cell proliferation, suggesting that the knockdown of FABP5 suppressed the growth of NB cells (Fig. 6i–k). Moreover, a pronounced decrease in cell number in the lower chamber was observed in the FABP5 knockdown groups, which indicated that loss of FABP5 inhibited NB cell migration and invasion (Fig. 6l,m). Overall, these results showed that FABP5 had tumorigenic effects on NB proliferation, migration and invasion.

### Over-expression of FABP5 reverses the tumor inhibitory effects of NBASP

**Fig. 7:**
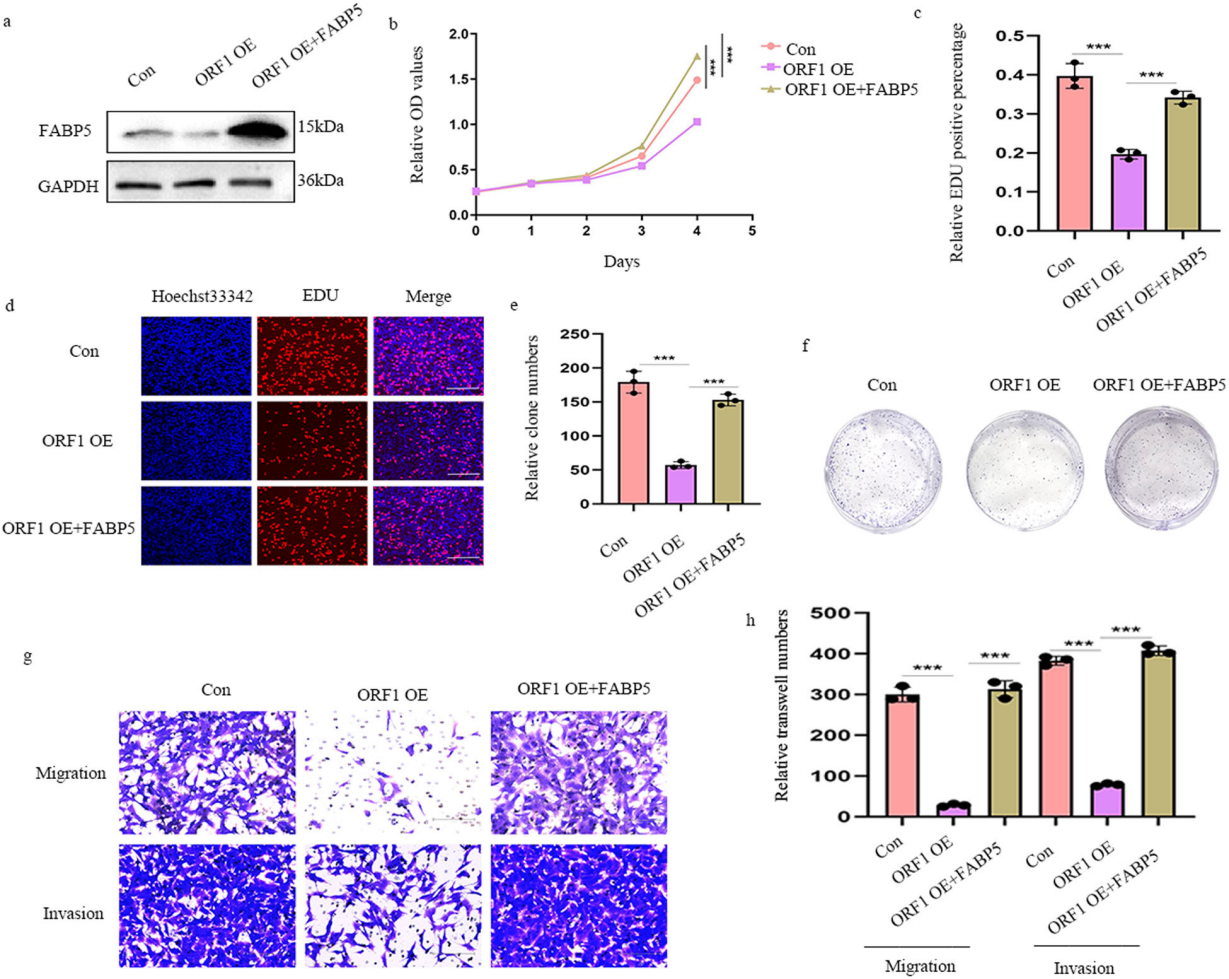
Over-expression of FABP5 restores the tumor inhibitory effects of NBASP. **a** Western blotting showed the restored efficiency; **b** In the CCK8, over-expression of NBASP suppressed the growth of cells, however, over-expression of FABP5 in NBASP stably transfected cells facilitated the impaired viability; **c**–**f** The EdU (**c** and **d**) and colony formation (**e** and **f**) assays further validated and quantified the proliferative effect of FABP5 in NBASP over-expressed NB cells, magnification of EdU, ×200, scale bar, 300 μm; **g** and **h** The effects on migration and invasion of NBASP in NB cells was restored by FABP5, as shown by transwell experiments, magnification, ×100, scale bar, 300 μm. Results in (**a**–**h**) are mean ± SD (*n* = 3). Statistical analysis was done by Student’s *t* test. (^***^p < 0.001).

NBASP has been shown anti-cancer roles in NB. To ascertain whether FABP5 restored the tumor suppressor effect of NBASP, it was over-expressed in cells stably transfected for over-expression of ORF1. We detected FABP5 expression in the restored cell lines (Fig. 7a). The CCK-8 assay indicated that NBASP encoded by ORF1 suppressed the proliferation of cells, however, FABP5 facilitated the impaired viability (Fig. 7b). In a similar manner, EdU and colony formation assays further confirmed the proliferative effects of FABP5 in NBASP over-expressed NB cells (Fig. 7c–f). Following these findings, the anti-migration and invasion effects of NBASP in NB cells was rescued by FABP5, as shown by transwell experiments (Figs. 7g,h). Based on these results, we proposed that NBASP played an anti-tumor role through negatively regulated FABP5.

### NBASP reduces tumor growth via the FABP5-mediated MAPK pathway

**Fig. 8:**
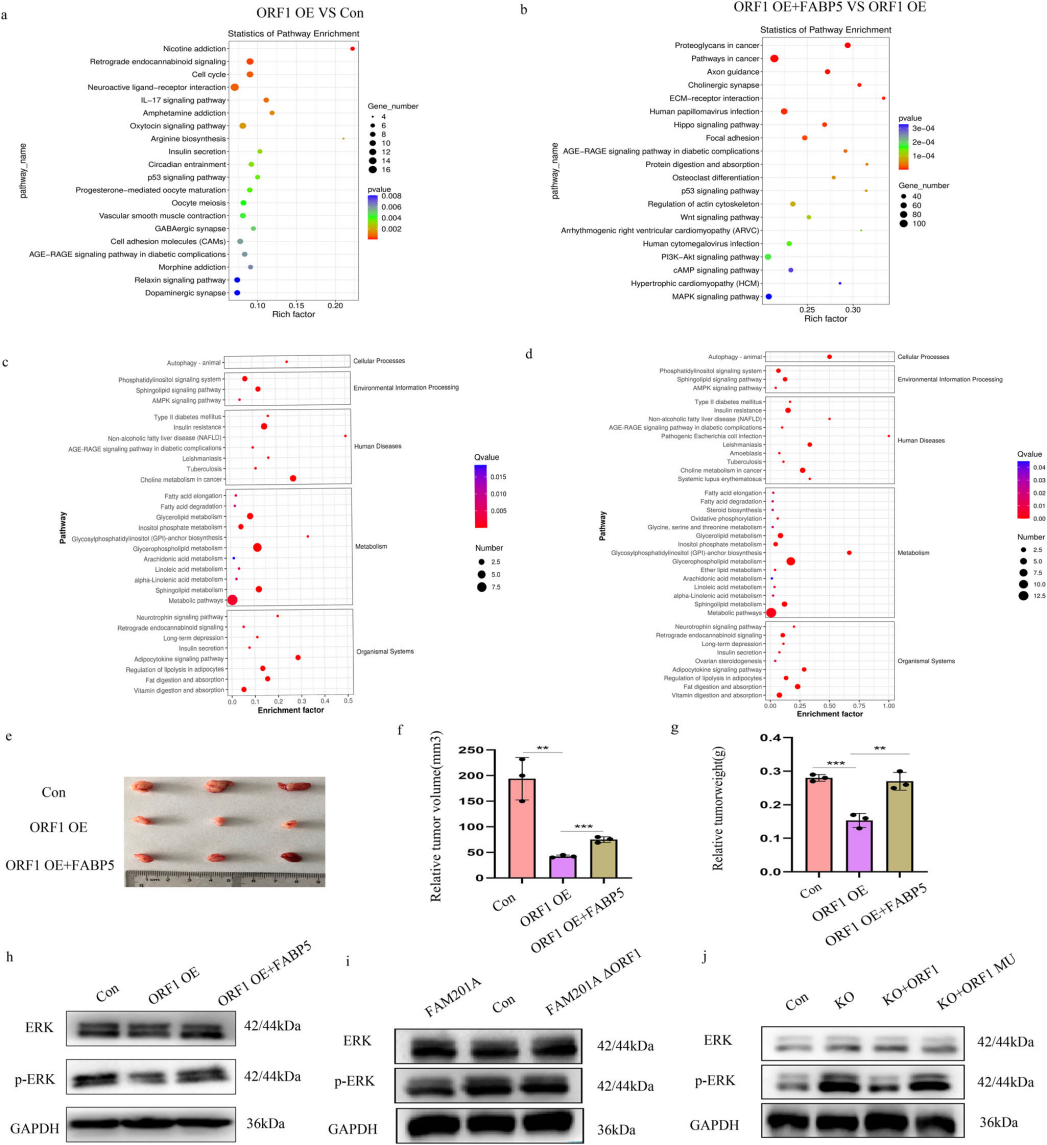
NBASP reduces tumor growth via the FABP5-mediated MAPK pathway. **a**–**d** Kyoto Encyclopedia of Genes and Genomes of RNA-seq (**a** and **b**) and lipid metabolomics (**c** and **d**) were shown in ORF1 over-expressed cells, ORF1 over-expressed+FABP5 over-expressed cells, and control cells; **e**–**g** General appearance (**e**), volumes (**f**), and weights (**g**) of subcutaneous tumor tissues from nude mice; **h** Western blotting showed that ORF1 inhibited MAPK signaling and FABP5 re-activated MAPK signaling; **i** Western blotting showed ERK and p-ERK protein levels in FAM201A over-expression, FAM201A over-expression with ORF1 depletion, and control groups; **j** Western blotting showed ERK and p-ERK protein levels in FAM201A knockout, ORF1 restore, ORF1 mutation at start codon(ATG to ATT) restore, and control groups. Results in (**e**–**j**) are mean ± SD (*n* = 3). Statistical analysis was done by Student’s *t* test.(^**^p < 0.01, ^***^p < 0.001).

As FABP5 is a fatty acid-binding protein, it can promote tumors by affecting lipid metabolism. To better understand the underlying mechanism involved in the progression of NB cells, we performed RNA-seq and lipid metabolomics studies in ORF1 over-expression and FABP5 rescued cell lines, which showed that NBASP was involved in numerous biological processes and several signaling pathways, such as, MAPK signaling (Fig. 8a–d). Furthermore, an in vivo assay showed NBASP inhibited tumor growth, and that FABP5 restored anti-cancer activity (Fig. 8e–g). Further western blotting showed that NBASP inhibited MAPK signaling, while FABP5 could activate MAPK signaling (Fig. 8h). Moreover, FAM210A inactivated MAPK signaling while when ORF1 deletion from FAM201A it lost this function (Fig. 8i). Consistently, FAM201A knockout activate MAPK signaling and ORF1 could repress the activation, which suggested that influence of FAM201A on MAPK signaling pathway was mediated by ORF1-encoded NBASP (Fig. 8j). Overall, ORF1 was the essential element of FAM201A, and it encoded NBASP functioned as tumor suppressor.

## Discussion

Due to clinical and genetic heterogeneities, NB still accounts for 15% of pediatric cancer deaths, despite decades of therapeutic advances, indicating the importance to identify new molecular pharmacological targets^3^. Several lncRNA studies contributed new concepts in improving both prognoses and therapeutic approaches in many diseases, particularly cancer^21^. Accumulating evidence indicated that the involvement of lncRNAs during malignancy and NB initiation emphasized their potentials as indicators of bad/good prognoses. Although most of these related studies focused on the molecular mechanisms by which the lncRNAs acted, with progress of bioinformatics and translation-omics studies, investigators have found that some lncRNAs encoded functional micropeptides, which provided us with a new perspective in understanding the role of lncRNAs in tumors^22–25^. In the present work, we identified NBASP encoded by FAM201A, which inhibited the development of NB, and which may ultimately provide a new and highly effective target for NB patients.

In previous studies related to FAM201A, the expression of FAM201A showed a relatively consistent up-regulation trend in several tumors, including lung adenocarcinoma, triple-negative breast cancer, and hepatic cancer cells^15,16,18,19,26^. It promoted their abilities to proliferate and invade, and even mediate the radio-sensitivity of non-small-cell lung and esophageal squamous cell cancers^17,27^. Although there are still few studies on FAM201A, we have found from existing studies that most of the effects of FAM201A on tumors were through regulating micro-RNAs, which directly or indirectly affected the expressions of target genes^15–18,26,27^. In the present study, we showed that FAM201A was down-regulated in NB cell lines and clinical tumor tissue samples, and closely associated with proliferation and metastases of NB cells, indicating its putative role as an anti-oncogene. Furthermore, we identified and characterized a functional small peptide, NBASP, encoded by FAM201A. It functioned as a tumor suppressor by encoding NBASP, rather than as a lncRNA. Moreover, we showed that NBASP suppressed NB cell proliferation, invasion, and migration in vivo and in vitro.

To identify the mechanism mediated by NBASP, we combined Co-IP and loss-and-gain functional experiments, and found that NBASP bound FABP5 when participating in its anti-tumor effect. The synthesis of fatty acids occurs in almost all tumors, because they require lipids, both as membrane components and as signaling molecules involved in stress responses, cell survival, cell death, and metastasis^28,29^. Notably, NB was highly reliant on fatty acid oxidation for energy production, and inhibition of β-oxidation resulted in reduced cell and tumor growth, especially for MYCN-amplified cells and tumors^30^. Fatty acid-binding protein 5 (FABP5) is an intracellular lipid-binding protein that binds with high affinity to medium and long-chain fatty acids, and plays an essential role in fatty acid uptake, transport, and metabolism. Previous studies have shown FABP5 up-regulation in various tumors^28^, indicating that FABP5 is an oncogene that enhances the proliferation, invasiveness, survival, and inflammation in liver cancer^31^, cervical^32,33^, colon cancer^34^, renal cancer^35^, and breast cancer cells^36,37^. Consistent with these results, we also showed that FABP5 promoted NB development and progression.

Furthermore, up-regulation of FABP5 in NBASP over-expressing cells reversed the anti-tumor effect of NBASP. RNA-seq results further suggested that the signaling pathway inactivated by over-expression of NBASP involved MAPK signaling, which was mediated by FABP5. Previous studies reported that active MAPK was usually associated with increased malignancy during the progression of NB^38–40^. Although we showed that FABP5 interacted with NBASP and inhibited the MAPK pathway, how these two proteins cooperatively contribute to MAPK signaling still needs further study.

In conclusion, our findings showed that NBASP was an anti-tumor small protein encoded by FAM021A. Mechanistically, functional investigation revealed that NBASP interacted with FABP5 and reduced its expression via ubiquitin proteasome pathway, to inhibit progression of NB tumorigenesis via MAPK signaling. Previous studies have suggested that peptides could be delivered to tumor cells through nanoparticles or recombined with adenovirus and injected into patients as anti-cancer therapies^41^. Moreover, these peptides are expected to be used as promising anti-cancer drugs, which suggests potential clinical uses of NBASP in NB therapy.

## Materials and methods

### Human cell culture and NB samples

Human NB cell lines, CHLA15, CHLA136, SK-N-SH, SH-SY5Y, and SK-N-AS, were maintained in high glucose DMEM (Hyclone, Logan, UT, USA), while SK-N-BE(2)C and SK-N-BE(2) cells were maintained in Ham’s F 12 nutrient medium (Gibco, Gaithersburg, MD, USA). Among these cell lines, SK-N-BE(2) and SH-SY5Y was purchased from the Cell Bank of the Chinese Academy of Science (Shanghai, China) and other cells were kind gifts from Professor Kai Li of Children’s Hospital of Fudan University. Both media were supplemented with 10% fetal bovine serum (FBS; Gibco) and penicillin-streptomycin solution (Gibco), and cells were cultured at 37 °C in a humidified atmosphere containing 5% CO_2_. Our study collected primary NB tumor tissues and peritumoral tissues from the Children’s Hospital of Fudan University. All patients with NB were diagnosed by pathology department and did not receive any treatment prior to surgery. Informed consent was obtained from each patient or legal guardian. And the methods were performed in accordance with relevant guidelines and regulations and approved by Committee of Children’s hospital of Fudan University.

### RNA extraction and quantitative real-time PCR(Q-PCR)

Total RNA was isolated from tissues and cells using TRIzol reagent (Takara, Shiga, Japan). PrimeScript RT Reagent Kit with gDNA Eraser (Yeasen, Shanghai, China) was used for reverse transcription. Real-time PCR was conducted with a Heff UNICON Universal Blue qPCR SYBR Green Master Mix (Yeasen). Short hairpin RNA of FABP5 and guide RNA of FAM201A (Supplementary Table 1) was designed and constructed by Genomeditech(Shanghai, China). Relative mRNA expression was calculated using the ΔΔCt method, and GAPDH was used as the internal control for normalization. Detailed primer sequences are listed in Supplementary Table 2.

### Cell viability assay

The procedures for testing cell viability were performed using a Cell Counting Kit-8 (CCK8; Yeasen), a 5-ethynyl-20-deoxyuridine (EdU) assay kit (Ribobio, Guangzhou, China), and a colony formation assay. SK-N-BE (2) and SH-SY5Y cells were seeded into 96-well plates at a density of 4 × 10^3^ cells and 3 × 10^3^ cells per well, respectively. CCK-8 (10 µL) was added to cells and incubated for 2 h at 37 °C, then the absorbance was measured at 450 nm daily for 4 consecutive days. For EdU measurements, the cells were seeded into 96-well plates at a density of 1 × 10^5^ cells per well, incubated in 50 µM EdU buffer for 2 h at 37 °C, fixed with 4% paraformaldehyde (PFA) for 0.5 h, and permeabilized with 0.1% Triton X-100 for 20 min. Next, EdU solution was added to cultures, followed by staining nuclei with Hoechst33342, and the results were visualized using a fluorescence microscope (Thermo Fisher Scientific, Waltham, MA, USA). For the colony formation assay, 2 × 10^3^ cells and 1.5 × 10^3^ cells per well were seeded into six-well plates and cultured for about 2 weeks. After 14 d of incubation, the plates were washed with phosphate-buffered saline (PBS), fixed in 4% PFA for 15−20 min, and stained with 0.1% crystal violet solution for 10–15 min for further analyses.

### Cell migration and invasion assays

Migration and invasion assays were performed with 24-well plates fitted with 8 µm pore size transwell filters, with or without precoated diluted matrigel (1:5; Becton Dickinson, Franklin Lakes, NJ, USA). SH-SY5Y cells at a cell density of 2.5 × 10^5^ (migration) and 5 × 10^5^ (invasion), and SK-N-BE (2)C cells at a density of 2 × 10^5^ (migration) and 4 × 10^5^ (invasion), diluted in serum-free medium, were placed in the upper chamber and medium containing 30% FBS added to the lower chamber. After incubating for 48 h at 37 °C, the cells on the underside of the membrane were fixed with 4% PFA for 15 min and stained with 0.1% crystal violet solution within 20 min for further analysis. Penetrating cells from five random fields were counted using a microscope.

### Western blotting

Tissues and cells were lysed in ice-cold NP40 lysis buffer (Beyotime, Nantong, China), then mixed with the protease inhibitor, phenylmethylsulfonyl fluoride (Beyotime). Protein concentrations were quantified with the Bradford assay using a Coomassie Brilliant Blue G250 reagent kit (Beyotime). After denaturation of the extracted protein by boiling at 95 °C for 10 min, equal amounts of protein were resolved on an SDS-PAGE gel (New Cell & Molecular Biotech, Suzhou, China) and transferred electrophoretically onto polyvinylidene fluoride membranes. Membranes were then blocked with 8% skimmed milk for 1 h, then incubated with antibodies at 4 °C overnight. The next day, after washing with TBST three times, the membranes were incubated with horseradish peroxidase-conjugated secondary antibodies for 1 h at room temperature. Using GAPDH as a control, western blotting signals were obtained from the imaging system using an enhanced chemiluminescent reagent kit (New Cell and Molecular Biotech). All antibodies used in this study are listed in Supplementary Table 3. The anti-NBASP polyclonal antibody was raised against rabbits and made by Hangzhou Hua’an Technology (Hangzhou, China).

### Immunofluorescence staining

Cells were cultured on coverslips and fixed with 4% PFA for 15 min, permeabilized with 0.5% Triton X-100 for 15 min, washed with PBS, followed by blocking in 5% bovine serum albumin for 1 h, then incubated with anti-FLAG antibody (1:5,000 dilution) at 4 °C overnight. Immunofluorescence was visualized using a fluorescence microscope.

### Co-immunoprecipitation (Co-IP) and mass spectrometry

Co-IP was conducted using a Dynabead Protein G Immunoprecipitation Kit (Thermo Fisher Scientific), following the manufacturer’s protocols. Whole cell lysates from cells transfected with the ORF plasmid and FLAG empty vector were prepared using lysis buffer. Co-IP was performed with anti-FLAG and anti-HA antibodies (Cell Signaling Technology, USA). SDS-PAGE resolved the samples, and differential bands were visualized using the silver staining method. Proteins represented by the differential bands were excised and further analyzed by mass spectrometry.

### Tumorigenicity in nude mice

For animal assays, 2 × 10^6^ NB cells were injected into BALB/c female nude mice (5-weeks-old) in 0.1 mL PBS. After 1 month, mice were killed, and the tumor tissues were collected and measured for volume and weight. The animal assay was approved by the Institutional Animal Care and Use Committee of Nanjing Medical University.

### Statistics and reproducibility

All results are presented as the mean ± SD, and the data were analyzed using GraphPad Prism 9.0 software. We assessed the significance between two groups using the Student’s *t* test. A value of *P* < 0.05 was considered significant.

### Reporting summary

Further information on research design is available in the Nature Portfolio Reporting Summary linked to this article.

## Supplementary information

Supplementary Information

Description of Additional Supplementary Files

Supplementary Data 1

Reporting Summary

## Data Availability

All data generated or analyzed during this study are included in this published paper and its supplementary information files. The source data for all the graphs and charts in the main figures are available as Supplementary Data 1 and any remaining information can be obtained from the corresponding author upon reasonable request. Raw gel images could be found in Supplementary Information.
