# Supplementary material for: FAM201A encodes small protein NBASP to inhibit neuroblastoma progression via inactivating MAPK pathway mediated by FABP5
Source: Commun Biol. 2023 Jul 12;6:714. doi: 10.1038/s42003-023-05092-7 (PMC10338675; doi:10.1038/s42003-023-05092-7)
Supplement: Supplementary file 1 — Supplementary Information [file 42003_2023_5092_MOESM1_ESM.pdf]

Supplementary Table1 Target sequence of FABP5/FAM201A

Gene names	Target sequence(5’-3’)
FABP5 sh	GGCGCCTGGTGGACAGCAAAG
FAM201A sgRNA NC	ACGGAGGCTAAGCGTCGCAA
FAM201A sgRNA 1	CTGGCTGCGGCCAAGCCAGG
FAM201A sgRNA 2	GATGGCTGGCGCTCCCAGCC
FAM201A sgRNA 3	CCCTCCGCAGCCACCGGGGA
FAM201A sgRNA 4	AGAGTCTCGCTCTTTCGCCC
FAM201A sgRNA 5	CCCATAAATGTATGCAATTG
FAM201A sgRNA 6	AATGAGGAATGACTACATCG

Supplementary Table2 Primers of genes

Gene names	Sequence(5’-3’)
FAM201A forward	TCTCTGATGGGAGCCTCTTTA
FAM201A reverse	CAAGCCACAGACGGAGAAA
GAPDH forward	GGAGCGAGATCCCTCCAAAAT
GAPDH reverse	GGCTGTTGTCATACTTCTCATGG

SupplementaryTable3 Antibody information

Antibody	Company	Catalogue	Dilution ratio
GAPDH	Proteintech	60004-1-Ig	1：5000
β-Actin	Proteintech	60008-1-Ig	1：5000
FLAG	Proteintech	66008-4-Ig	1：5000
ERK	CST	4695S	1：1000
p-ERK	CST	4370S	1：1000
NBASP	Hua’an	/	1：1000
FABP5	CST	39926T	1：1000
Ubiquitin	Proteintech	10201-2-AP	1：1000
Goat Anti-Mouse IgG	CWBIO	CW0102S	1：5000
Goat Anti-Rabbit IgG	CWBIO	CW0103S	1：2000

Supplementary Figures of western blot

Figure 3c

FLAG

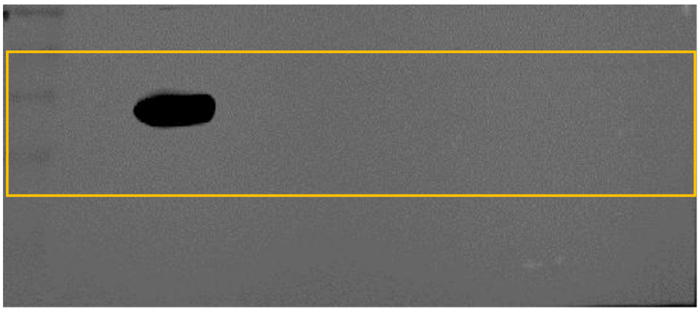

β-Actin

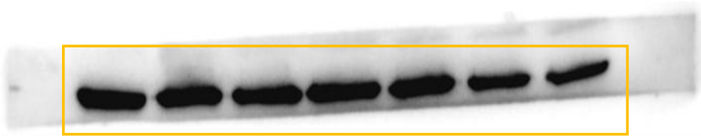

Figure 3f

FLAG

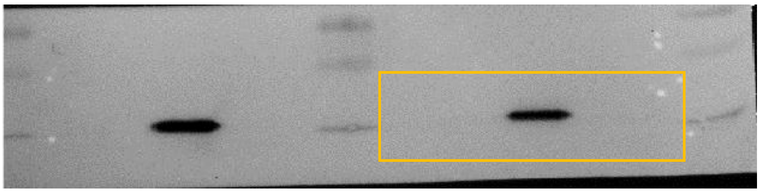

β-Actin

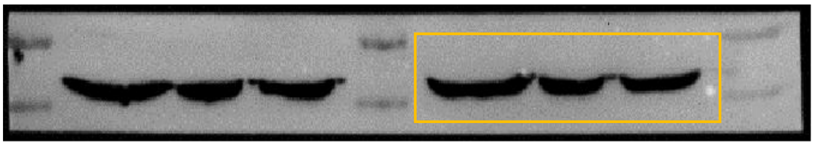

Figure 5a

NBASP

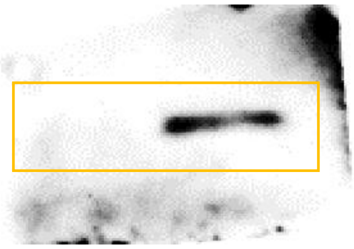

GAPDH

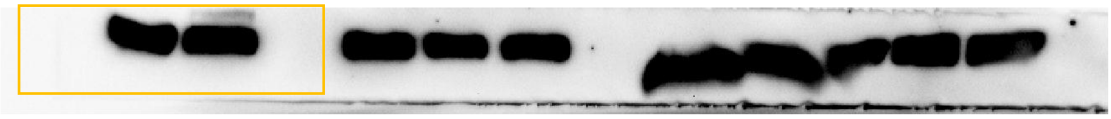

NBASP

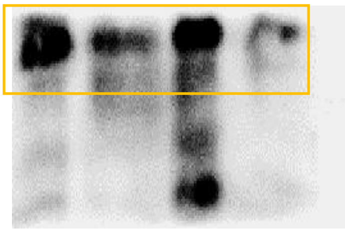

GAPDH

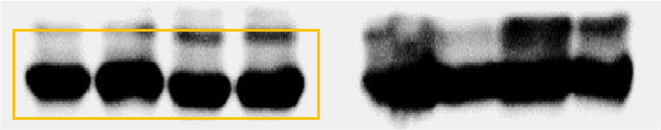

Figure5b

NBASP

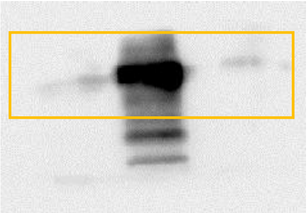

GAPDH

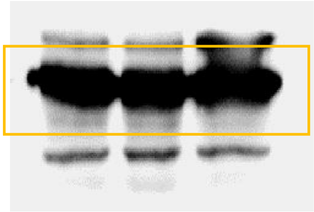

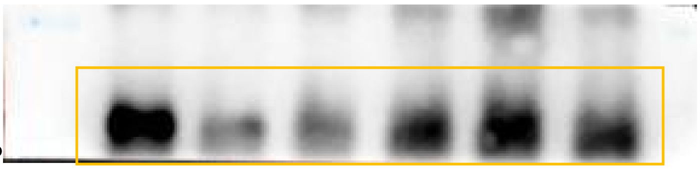

NBASP

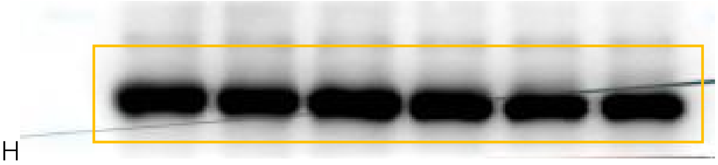

GAPDH

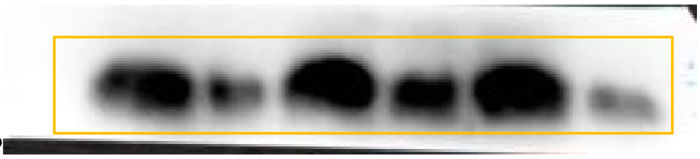

NBASP

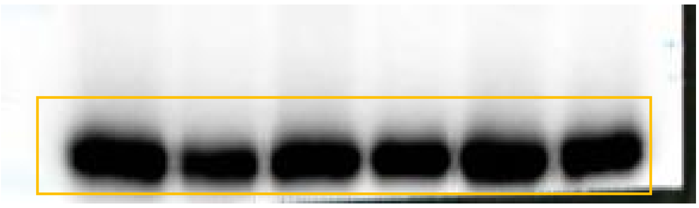

GAPDH

Figure5e

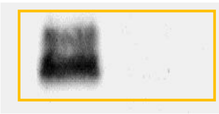

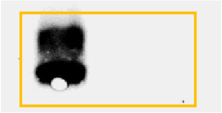

FLAG+HA

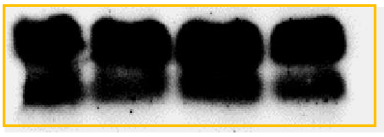

FLAG+HA

Figure5f

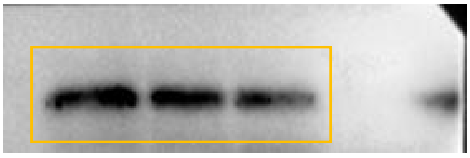

FABP5

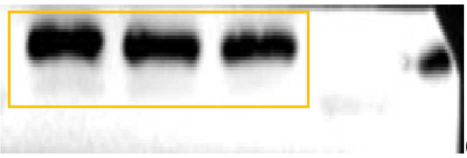

GAPDH

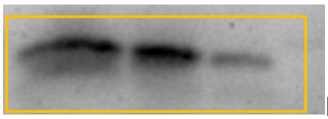

FABP5

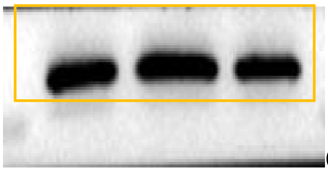

GAPDH

Figure5g

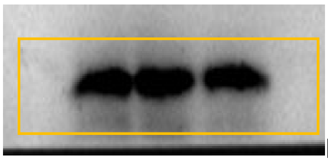

FABP5

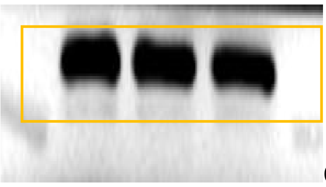

GAPDH

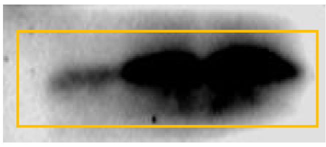

FABP5

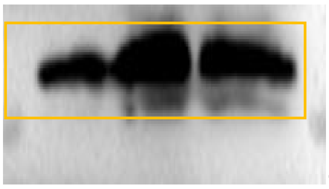

GAPDH

Figure5h

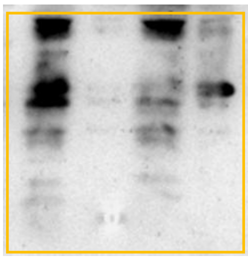

Ub

Figure5i

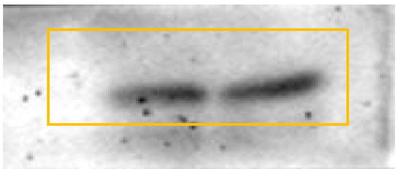

NBASP

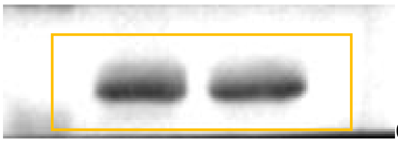

GAPDH

Figure6a

FABP5

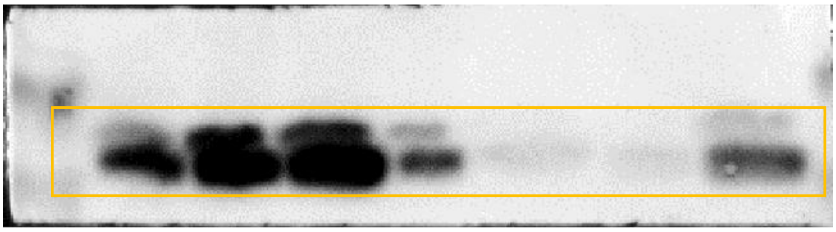

GAPDH

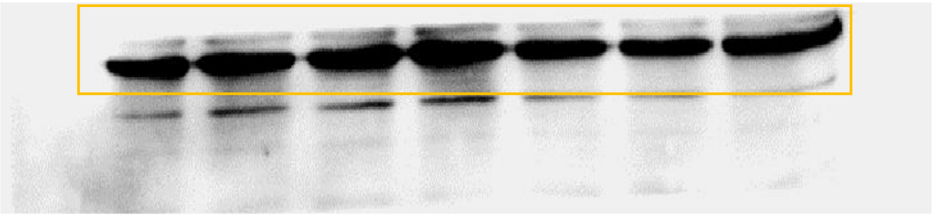

Figure6c

FABP5

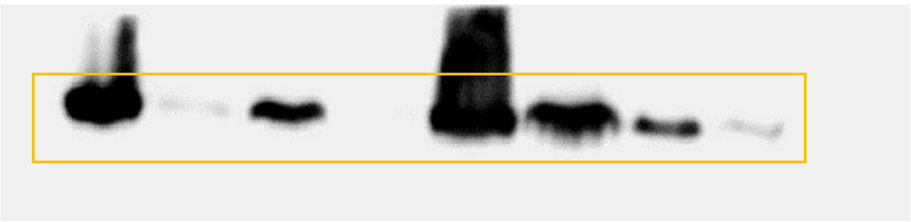

GAPDH

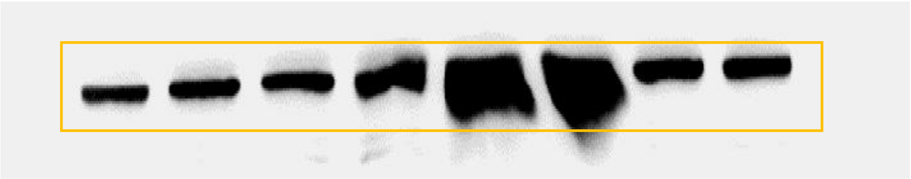

FABP5

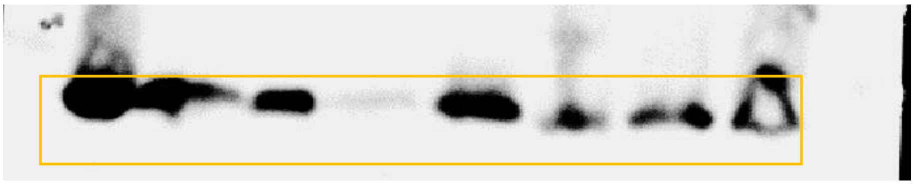

GAPDH

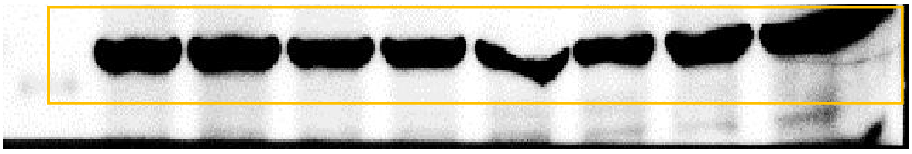

Figure6e

FABP5

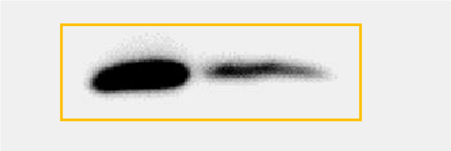

GAPDH

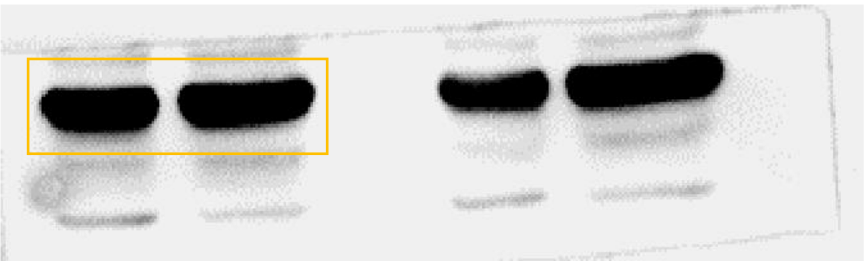

FABP5

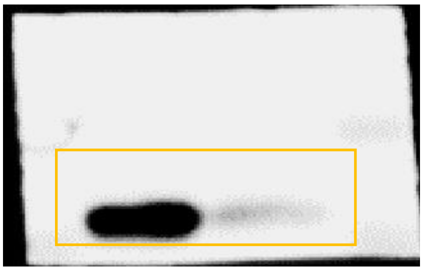

GAPDH

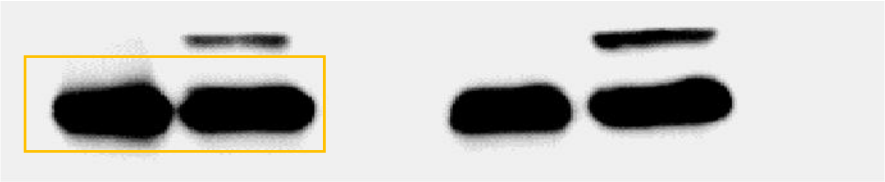

Figure6g

FABP5

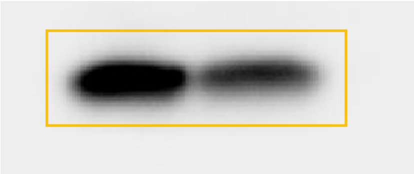

GAPDH

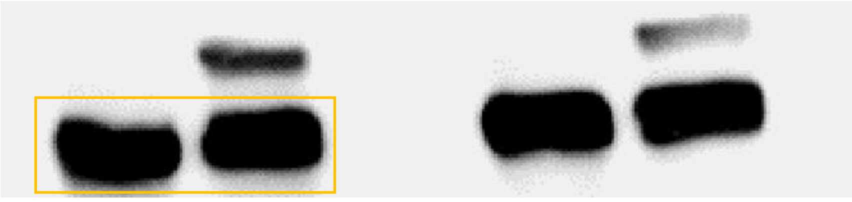

Figure7a

FABP5

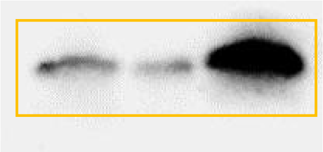

GAPDH

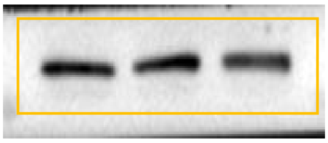

Figure8h

ERK

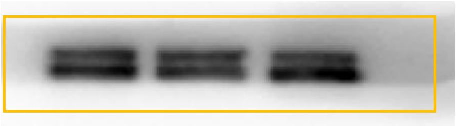

P-ERK

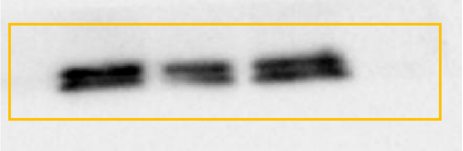

GAPDH

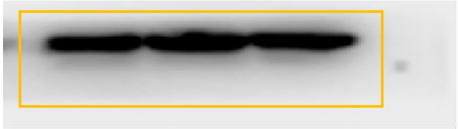

Figure8i

ERK

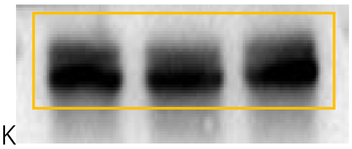

P-ERK

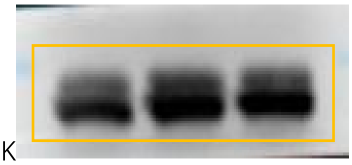

GAPDH

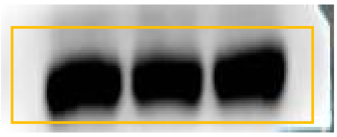

Figure8j

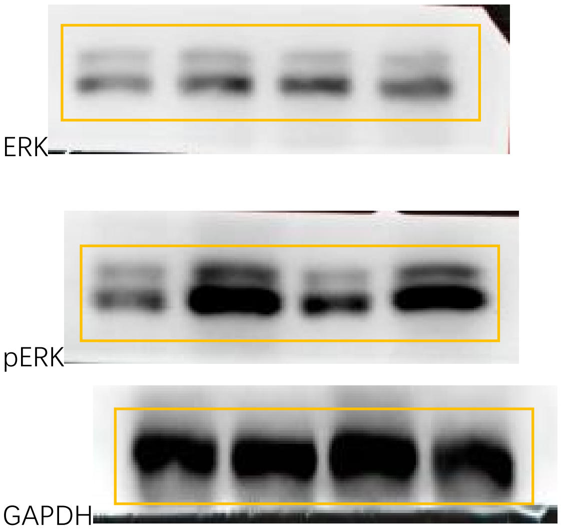

ERK

pERK

GAPDH
