# Supplementary material for: FAM201A encodes small protein NBASP to inhibit neuroblastoma progression via inactivating MAPK pathway mediated by FABP5
Source: Commun Biol. 2023 Jul 12;6:714. doi: 10.1038/s42003-023-05092-7 (PMC10338675; doi:10.1038/s42003-023-05092-7)
Supplement: Supplementary file 2 — Description of Additional Supplementary Files [file 42003_2023_5092_MOESM2_ESM.pdf]

Description of Additional Supplementary Files

File name: Supplementary Data 1
Description: Supplementary targets, primers, antibodies, and western blot bands.
